# Exposure to delayed visual feedback of the hand changes motor-sensory synchrony perception

**DOI:** 10.1007/s00221-012-3081-0

**Published:** 2012-05-24

**Authors:** Mirjam Keetels, Jean Vroomen

**Affiliations:** Department of Medical Psychology and Neuropsychology, Tilburg University, Tilburg, The Netherlands

**Keywords:** Temporal recalibration, Motor-sensory synchrony, Simultaneity judgment task, Delayed visual feedback

## Abstract

We examined whether the brain can adapt to temporal delays between a self-initiated action and the naturalistic visual feedback of that action. During an exposure phase, participants tapped with their index finger while seeing their own hand in real time (~0 ms delay) or delayed at 40, 80, or 120 ms. Following exposure, participants were tested with a simultaneity judgment (SJ) task in which they judged whether the video of their hand was synchronous or asynchronous with respect to their finger taps. The locations of the seen and the real hand were either different (Experiment 1) or aligned (Experiment 2). In both cases, the point of subjective simultaneity (PSS) was uniformly shifted in the direction of the exposure lags while sensitivity to visual-motor asynchrony decreased with longer exposure delays. These findings demonstrate that the brain is quite flexible in adjusting the timing relation between a motor action and the otherwise naturalistic visual feedback that this action engenders.

## Introduction

While interacting with the external world, our brain is provided with a massive amount of information that is processed by different neural mechanisms. For example, the clapping of the hands is initiated by the motor system, but subsequently, visual, auditory, and tactile information is provided and processed. Despite that motor planning and the subsequent motor execution vary in time for different actions, and different sensory modalities process information from a common event at different speeds (Fain 2003), people still experience the sensory consequences of their actions as causal and as simultaneous with the actions. Though, in artificial situations like a teleconference or a telesurgery in which the auditory and/or visual information can be noticeably delayed, people may experience incoherence between their motor actions and the sensory consequences. Here, we examined to which extent the brain can adapt to these temporal disturbances of motor-sensory events by exposing participants to delayed, but otherwise naturalistic visual feedback of their own body movements. The question was whether participants would recalibrate their sense of motor-visual synchrony of what, at first sight, would seem to be a rigid timing relation.

Initial studies on temporal recalibration have demonstrated that participants do indeed adapt to small asynchronies between artificial audio-visual stimuli like flashes and beeps (Fujisaki et al. 2004; Vroomen et al. 2004). For example, participants who were exposed to a flash occurring ~100 ms after a beep were in simultaneous test trials more likely to say that the flash occurred *before* the beep. Ever since, this so-called cross-modal temporal recalibration effect (i.e. TRE) has been reported many times in different situations and in different modalities (see Vroomen and Keetels 2010 for reviews; Keetels and Vroomen 2012). In principle, though, there may be nothing implausible about small temporal delays between the senses, given that we are exposed to visual and auditory stimuli in all sorts of temporal configurations due to differences in air-transduction and neural processing times (Fain 2003). The order of motor actions and their sensory feedback, though, is much more constrained because sensory feedback is normally expected to occur only *after* motor actions are initiated. As an example, you will only hear yourself speaking after you actually spoke the utterance. Yet, flexibility to motor-sensory timing relationships seems to be necessary, at least to some extent, because conduction times in sensory and motor pathways change due to, for example, differences in retinal response times in different lighting conditions (Purpura et al. 1990), attention to specific modalities (i.e. prior entry: attention towards a specific modality can speed up processing time in the attended modality; Titchener 1908) or, on a longer time scale, due to limb growth (Campbell et al. 1981) or muscle decay.

For the visual-motor domain, several authors have reported that temporal recalibration does indeed occur and that it can actually change the causal relationship between a motor action and the associated *artificial* sensory feedback (i.e. like the movement of a cursor or a visual flash; Stetson et al. 2006; Cunningham et al. 2001; Sugano et al. 2010; Pesavento and Schlag 2006; Heron et al. 2009; Stekelenburg et al. 2011). Stetson et al. (2006), for example, exposed participants to a fixed delay between a self-initiated key-press and a subsequently delivered flash and reported that flashes appearing at unexpectedly short delays were often perceived as occurring *before* the motor action, thus demonstrating a reversal of ‘cause-before-effect’. Sugano et al. (2010) also examined motor-sensory recalibration and explored whether it is the sensory or motor event that is shifted in time. Participants were exposed to a 150 ms lag between a finger tap and a flash or tone pip, and immediately thereafter, they performed a temporal order judgment (TOJ) task about the tap-feedback test stimulus. The modality of the feedback stimulus was either the same as the adapted one (within modal) or different (cross-modal). The results showed that the point of subjective simultaneity (PSS) was uniformly shifted in the direction of the exposed lag within and across modalities, indicating that the TRE of sensor-motor events is mainly caused by a shift in the motor component (though, see Stekelenburg et al. 2011 for effects in the visual ERPs).

At this stage, though, motor-visual temporal recalibration has only been studied with artificial sensory feedback, but not with naturalistic feedback. There are several reasons why the causal relationship between a motor action and the resulting natural sensory feedback might be different when compared with artificial sensory feedback. Most importantly, delays to natural feedback are far less variable than delays to artificial feedback. For example, when watching your own hand reaching for an object, it is only the relatively stable visual sensory transduction time that might cause a delayed perception of the limb’s movement. For artificial feedback, though, delays can be quite large and variable due to for example, technical constraints or operating system delays. For example, the time that passes between a finger touching a keyboard and a letter appearing on a computer screen differs substantially because the sensitivity of keys on different keyboards varies widely. Another potentially relevant factor is that there may be more *intentional binding* between a voluntary movement and natural rather than artificial feedback. One consequence of intentional binding is that the interval between a voluntary action and its outcome is perceived to be shorter than the interval between a physically similar involuntary movement (Engbert et al. 2008; Haggard et al. 2002b; Moore et al. 2009; Buehner and Humphreys 2009). Intentional binding thus can diminish the perceived lag between an action and its outcome, and this may prevent temporal recalibration to occur simply because no timing error is detected.

One study that hints for an adaptation effect to delayed naturalistic *auditory* feedback was performed by Katz and Lackener (1977). In their study, participants were exposed to motor-sensory temporal disturbances by delaying auditory feedback (DAF; Lee 1950a, b, 1951) while performing speech tasks (i.e. reading word lists, short sentences, and a prose passage). The authors found that DAF had a disturbing effect directly after introducing the delay (i.e. high error rate and slow speech), but after being exposed to DAF for a few minutes, the disturbing effect declined. The authors concluded that participants had adapted to DAF in speech, and the reduction of errors might be due to the recalibration of motor-sensory timing. Alternatively, though, participants might also have learned to ignore the disturbing sounds. Given that the authors did not directly measure perceived motor-sensory timing, it remains unclear whether motor-sensory timing was recalibrated after exposure to DAF.

**Fig. 1:**
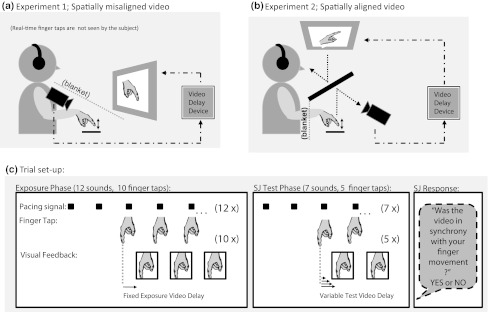
**a** Experiment 1: participants tapped their *right* index finger on the table while seeing their own finger in real time (0 ms delay) or delayed (40, 80, or 120 ms) on a monitor in front of them. **b** Experiment 2: participants tapped their *right* index finger while seeing their own finger, via a double-sided mirror at the location of their hand. **c** Each trial consists of an exposure phase and test phase, in which participants tapped 10 and 5 times, respectively, while the visual feedback of their finger movements was artificially delayed and played back on a monitor in front of them. Participants judged whether the video of the last 5 taps was synchronous or asynchronous (SJ task) relative to their movements

In the present study, we used finger taps as a voluntary action while the visual feedback consisted of an online video recording of that movement from the perspective of the actor, played back via a monitor with an adjustable delay. In Experiment 1, the monitor was placed in front of the participant at ~25 cm from the hand, which implied that the location and orientation of the seen and real hand did not match. In Experiment 2, we further increased the naturalness of the situation by aligning the orientation and location of the virtual and real hand. A single trial consisted of a short-exposure phase immediately followed by a test phase. During exposure, participants tapped their index finger while seeing the movement of the finger via the monitor (see Fig. 1a, b for Experiment 1 and Experiment 2, respectively). The normal synchrony between the motor action (M) and the accompanying visual information (V) was modified by delays in the video (varying from ~0 to ~120 ms; the shortest delays are hardly noticeable, the longer ones are). In the test phase, the delay of the video was again changed (varying from ~0 to ~160 ms), and participants decided whether the video was in- or out-of-synchrony with their finger taps (a simultaneity judgment task, SJ).^1^ We expected that motor-visual temporal recalibration would manifest itself as a shift in the point at which maximum simultaneity (the point of subjective simultaneity, PSS) between the motor act and the visual feedback was perceived.

## Experiment 1

### Method

#### Participants

Twenty-nine students (eight male, mean age: 21.4, 25 right-handed) from Tilburg University participated in return for course credits after giving informed consent. All had normal hearing and normal or corrected-to-normal seeing.

#### Stimuli and apparatus

Participants sat at a desk in a dimly lit and soundproof booth. Their right hand was positioned in front of them on a custom made touch pad (8 × 8 cm). A video camera (Conrad; KC-3800; CCD-Camera; PAL with 400 TV-lines at 50 Hz) was positioned on the table to record the participant’s finger movements (see Fig. 1a). The participant’s hand, touch pad, and camera were covered by a black cloth in order to prevent them from seeing their own real-time hand movements. Participants were instructed to rest their head on a chin rest and to look at a RGB monitor (PHILIPS, CM8533/00G) at about 45 cm viewing distance on which the real-time or delayed videos of their own hand movements were displayed. The video was delayed by dedicated hardware (Ovation Systems Ltd.; DelayLine; customized unit: up to 320 ms total delay, in 20 ms steps) connected between the video camera and the monitor. The intrinsic delay of this device was ~3 ms.^2^

To ensure that all participants received equal amounts of exposure and that they tapped at an equal speed, we presented an auditory train of click sounds that served as a pacing signal (ISI 750 ms; each click 10 ms duration; presented via headphones at 70 dB(A)). White noise was continuously presented via two speakers on each side of the monitor at 59 dB(A) to mask sounds produced by the taps.

#### Design

Two factors were varied: the exposure delay (0, 40, 80, or 120 ms video delay), and the delay of the test stimulus (0, 40, 80, 120, or 160 ms video delay). Half of the participants were tested with the 0 and 80 ms exposure delays (*N* = 15), and the other half were tested with 40 and 120 ms exposure delays (*N* = 14). The two exposure delays were split into two consecutive test sessions with at least a 1-h pause in between. The order of the exposure delays was counterbalanced across participants. The video delay in the test phase varied randomly. The whole experiment consisted of six blocks of 20 trials (three blocks per exposure delay), resulting in a total of 12 repetitions of each combination of test delay and exposure delay. Each block of 20 trials took about 8 min with a short break after each block. To acquaint participants with the procedures, experimental trials were preceded by a practice session of 6 trials with a 0 ms exposure delay.

#### Procedure

A single trial consisted of a short-exposure phase followed by a test phase (see Fig. 1c). During the exposure phase, participants were presented a sequence of 12 auditory clicks as a pacing signal and were instructed to tap along with the last 10 clicks on the touch pad with the index of their right hand. At the start of the exposure phase, the monitor was switched on, and depending on the particular exposure delay, a real-time video (0 ms video delay) or a delayed video (40, 80, or 120 ms video delay) of the participant’s hand was displayed. After the exposure phase, the monitor then switched off for 1.5 s (dark screen) after which the test phase started. During the test, participants were presented seven pacing clicks (750 ISI), and they tapped their right index finger along with the last five clicks. At the start of the test phase, the monitor was switched on again, and depending on the test delay, a real-time video (0 ms video delay) or a delayed video (40, 80, 120, or 160 ms video delay) of the participant’s actual finger movements was shown. After the last tap, the screen turned black, and a pure tone (70 dB(A), 440 Hz, 500 ms) was presented that indicated that participants had to decide whether the video that was seen during the test phase was synchronous with their finger movements or asynchronous. Participants made an unspeeded response by pressing one of the two buttons on a response box with their left hand. After the response, the exposure phase of the next trial started.

### Results

**Fig. 2:**
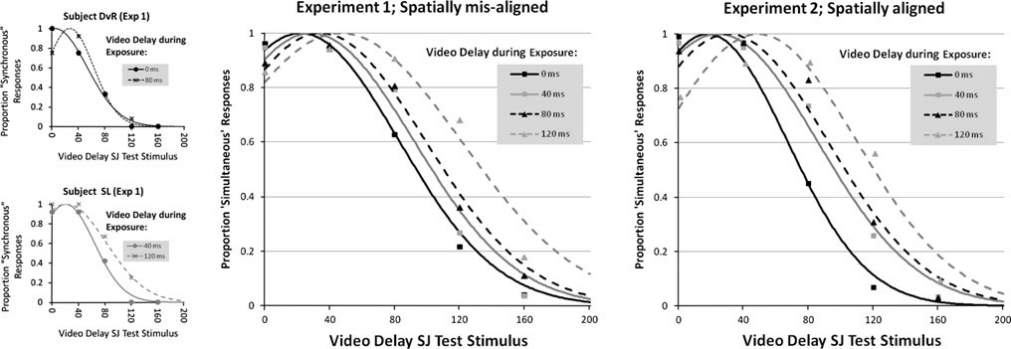
The proportion of ‘Synchronous’ responses as a function of the video delay of the test phase for Experiments 1 and 2. Different exposure delays are represented by *different lines*. In the *left panel*, the raw data and fitted functions of two representative participants of Experiment 1 are shown, and in *middle and right panel*, the averaged data over all participants are shown for Experiments 1 and 2, respectively

Trials of the training session were excluded from further analyses. The proportion of ‘synchronous’ responses was calculated for each participant and for each combination of exposure delay (0, 40, 80, 120 ms) and test delay (0, 40, 80, 120, 160 ms). For each of the obtained distributions of responses, an individually determined psychometric function was calculated by fitting the Gaussian normal distribution [exp(− (*x* − Mean)^2/(2*SD^2))] over the data using the ‘fminsearch’ function in Matlab 7.9 (MathWorks, Natick, MA, USA; the multidimensional unconstrained nonlinear minimization method). The average goodness of the fit was *R* ^2^ = 0.95. The mean of the resulting distribution is the point where simultaneity is maximal (the PSS), and the standard deviation (SD) is a measure of the sensitivity. Figure 2 displays an example of two representative participants and the averaged normal distributions. Table 1 shows the average PSSs and SDs at each exposure delay.

**Table 1:** For both Experiments 1 and 2, the mean PSSs and SDs after exposure to a 0, 40, 80, or 120 ms video delay during the exposure phase

	Video delay during exposure (ms)	PSS (S.E.M.)	SD (S.E.M.)
Experiment 1: spatially misaligned	0	23.4 (2.7)	57.1 (3.5)
	40	27.5 (3.3)	61.2 (3.2)
	80	35.1 (4.6)	62.7 (4.0)
	120	45.2 (4.1)	74.3 (4.9)
Experiment 2: spatially aligned	0	18.1 (1.5)	49.3 (2.5)
	40	26.1 (2.7)	59.4 (2.8)
	80	32.1 (2.4)	60.5 (2.7)
	120	47.0 (5.3)	62.1 (3.0)

Standard errors of the mean (S.E.M.) are reported in parenthesis

A mixed-linear model analysis with exposure delay (0, 40, 80, 120 ms) as a fixed factor was performed on both the PSSs and the SDs. For both measures, a significant effect of exposure delay was found: PSS: *F*(3,58) = 6.92, *p* = <.001, SD: *F*(3,58) = 3.72, *p* = <.05. The effect of exposure delay on the PSS demonstrates that participants adjusted the perception of synchrony, as the most synchronous test stimulus gradually shifted with exposure delay: The PSSs were 23.4, 27.5, 35.1, and 45.2 ms after exposure delays of 0, 40, 80, and 120 ms, respectively. The effect of exposure delay on the SDs shows that sensitivity to motor-sensory asynchronies gradually declined when the exposure delay increased (SD: 57.1, 61.2, 62.7, and 74.3 ms after exposure delays of 0, 40, 80, and 120 ms, respectively). The best fitting linear function through the PSS values was as follows: PSS = (0.18* exposure delay) + 22.0 ms, which thus indicates that the PSS shifted about 18 % of the exposure delay. A similar linear function fitted over the standard deviation (SD = 0.13* exposure delay + 55.9), indicates that sensitivity declined with about 13 % of the exposure delay.

### Discussion

The most important result of Experiment 1 is that the PSSs were shifted in the direction of the exposure delays. This shows that there is flexibility in motor-sensory timing, even with naturalistic feedback whose timing relation with the motor event is usually rather fixed. We also found that sensitivity to visual-motor asynchrony (SDs) declined with longer delays. Although PSS shifts are a common finding in temporal recalibration studies (for reviews, see Vroomen and Keetels 2010; Keetels and Vroomen 2012), the decrease in sensitivity has only been reported in some studies (Navarra et al. 2005; Vatakis et al. 2008, 2007). One concept that might have affected the PSSs and SDs in the present study is the ‘feeling of agency’. Because the motor system includes specific mechanisms for predicting the sensory consequences of our own actions (Frith et al. 2000; Blakemore et al. 2000), it might be that body actions were less strongly felt as ‘owned’ when participants saw their own hand movements at a different location and orientation than expected. In Experiment 1, the virtual hand was located ~25 cm further than the real hand, and this might have led to a reduced feeling of agency. A reduced feeling of agency, in turn, might cause a reduction in the number of ‘synchronous’ responses. To further examine this, we replicated Experiment 1 with the locations of the real and virtual hand spatially aligned.

## Experiment 2

### Method

Stimuli and design were the same as in Experiment 1 with the following exceptions. Thirty-two new participants (eight male, mean age: 19.9, 29 right-handed) from Tilburg University were tested (*N* = 17 for 0 and 80 ms exposure delays, and *N* = 15 for 40 and 120 ms exposure delays). They sat at a desk with their right hand holding a wooden block (7 × 7 × 4 cm) in the middle of the table. A double-sided mirror was installed above the participant’s hand (see Fig. 1b for a schematic overview of the apparatus set-up). The video camera projected the image of the participant’s hand via the backside of the mirror. The angle of the mirror and camera were adjusted in such a way that the seen orientation and location of the arm and hand matched the real one. A black cloth was attached to the front edge of the mirror to prevent the participant from seeing their own real-time wrist and hand movements.

### Results

PSSs and SDs were calculated as in Experiment 1 (see Fig. 2 and Table 1). A mixed-linear model analysis with exposure delay (0, 40, 80, 120 ms) as a covariate was performed on both the PSSs and the SDs. For both measures, a significant effect of exposure delay was found: PSS: *F*(3,64) = 15.89, *p* = <.001, SD: *F*(3,64) = 4.89, *p* = <.005. The effect of exposure delay on the PSS demonstrates that participants adjusted their perception of synchrony as the most synchronous test stimulus gradually shifted with exposure delay: the PSSs were at 18.1, 26.1, 32.1, and 47.0 ms after exposure delays of 0, 40, 80, and 120 ms, respectively. The effect of exposure delay on the SDs shows that sensitivity to motor-sensory asynchronies gradually declined when exposure delay increased (SD: 49.3, 59.4, 60.5, and 62.1 ms after exposure delays of 0, 40, 80, and 120 ms, respectively). The best fitting linear function through the PSS values was as follows: PSS = (0.23* exposure delay) + 17.0 ms, thus indicating that the PSS shifted about 23 % of the exposure delay. A similar linear function fitted over the SDs (SD = 0.1* exposure delay + 51.7) indicates that sensitivity declined with about 10 % of the exposure delay.

#### Between experiments analysis

A mixed-linear model analysis with exposure delay (0, 40, 80, 120 ms) and Experiment (1 vs. 2) as fixed factors was performed on both the PSSs and the SDs. A significant overall effect of exposure delay was found on PSSs (*F*(3,122) = 21.14, *p* = <.001) and SDs (*F*(3,122) = 7.25, *p* = <.001). There was no difference in the PSSs between experiments (*F*(1,122) < 1), but SDs were larger (a 6.0 ms overall difference) when the location of the real hand and its visual feedback were misaligned (Experiment 1) rather than aligned (Experiment 2; *F*(1,122) = 6.83, *p* = <.01). For both measures, no interaction was found between experiment and exposure delay (PSS: *F*(3,122) < 1; and SD: *F*(3,122) = 1.15, *p* = .33), indicating that the spatial separation between the seen and real location of the hand did not affect the pattern of temporal recalibration.

To further analyse the data, we examined the proportion of synchronous responses at the SOA that—under normal circumstances—should be the most naturalistic, namely the 0-ms SOA. If lag adaptation entails a true shift of the PSS rather only than a widening of the window of acceptable asynchronies in the direction of the adapted lag (see Yarrow et al. 2011a), one may expect the 0-ms SOA test stimulus to become ‘unnatural’ after exposure to large delays because observers may start to experience ‘consequence-before-cause’. To test this, we ran a separate analysis on the number of synchronous responses at 0 ms SOA (the left-most portion of the raw data in Fig. 2) with exposure delay (0, 40, 80, 120 ms) as a fixed factor. This analysis showed that for the 0-ms test trials, the proportion ‘synchronous’ responses declined after exposure to longer delays (average proportion ‘synchronous’ responses were .98, .96, .91, .81 for 0, 40, 80, and 120 ms exposure delays, respectively, *F*(3,122) = 9.1, *p* = <.001), thus confirming that there was a true shift in the PSS.

## General discussion

We examined the effect of exposure to delayed naturalistic visual feedback on the perception of motor-visual synchrony. Participants tapped their finger on a surface while viewing a video of their taps at 0, 40, 80, or 120 ms delays. Following a short-exposure phase, perception of motor-visual synchrony was tested by asking participants to judge whether the video of their finger was delayed relative to their taps. The results demonstrated that exposure to delayed visual feedback shifted the point of maximal perceived synchrony. This shift was not affected by whether the location of the seen hand and real hand was spatially aligned or misaligned. This suggests that observers are quite flexible in what constitutes motor-visual synchrony. This is remarkable given that the timing of self-initiated actions and their natural visual feedback is usually fixed.

The finding that the PSS was shifted after exposure to naturalistic delayed feedback concurs with the previous findings of temporal recalibration in both the intersensory (see for reviews: Vroomen and Keetels 2010; Keetels and Vroomen 2012) and the motor-sensory domain (Stetson et al. 2006; Sugano et al. 2010; Heron et al. 2009) in which so far only artificial stimuli have been used. Apparently, even though we are less frequently exposed to delays of naturalistic visual feedback, the brain is still able to adapt to these changes in timing (see also Kopinska and Harris 2004 who showed that the brain corrects for changes in visual timing, which result from wearing light-attenuating dark glasses). This fits with the idea that the brain needs to be flexible, to some extent, in order to correct for (small) changes in the motor and the visual system like limb growth, muscle decay, or diseases like Parkinson that affect motor control. Presumably, sensory signals from a motor event (tactile/kinaesthetic information) and the visual feedback are remapped to preserve their perceived simultaneity because events appearing at a constant delay after motor actions are interpreted as consequences of those actions. The brain then recalibrates the timing relation so that it is consistent with the prior expectation that sensory feedback follows motor actions without noticeable delay.

Several mechanisms have been proposed for how temporal recalibration between the senses might be envisaged (see Vroomen and Keetels 2010 for a review on this topic). One possibility that fits with the observation that there was a shift of the PSS in the direction of the adapted lag is that the criterion for simultaneity between the corresponding modalities is adjusted in accord with the previous experience (a Bayesian approach; Ernst and Bulthoff 2004). Alternatively, though, it may also be that one modality (vision, touch, or motor information) is ‘shifted’ towards the other because the sensory threshold for stimulus detection in the adapted modality is adjusted (Navarra et al. 2009). For example, participants may have adopted a more conservative criterion for detecting when they moved their finger or touched the surface, or they may have adopted a more lenient criterion for detecting when they actually saw the visual motion of the finger. Yet another possibility is that the ‘window of temporal integration’ was widened due to the asynchronous exposure. This idea of widening of the temporal window is in line with the finding that SDs became larger (i.e. sensitivity became worse) when the exposure delays increased. Note, though, that this widening of the temporal window cannot account for the whole pattern of results because physically synchronous test stimuli (0-ms SOA) were perceived as *less* synchronous (not more) after exposure to long video delays. Note that these options do not mutually exclude each other, and several of them remain open. The current data do indicate, though, that for motor-visual recalibration there may not be a critical distinction between naturalistic and artificial feedback because both kinds of feedback induce a shift in the PSS. Further research is necessary, though, to compare them in a more direct manner.

It is noteworthy that there was a decrement in sensitivity, because this has not always been reported. Navarra et al. (2005) and Vatakis et al. (2008) tested audio-visual temporal recalibration using audio-visual speech stimuli and also reported a decline in sensitivity. Their observers had to monitor a continuous speech stream for target words that were presented either in synchrony with the video of a speaker or with a delayed video. Concurrently with the speech-monitoring task, participants performed an audio-visual temporal order judgement task (TOJ; Navarra et al. 2005; Vatakis et al. 2007) or an SJ task (Vatakis et al. 2008) on simple flashes and noise bursts that were overlaid on the video. The results showed that participants became in both tasks less sensitive to audio-visual temporal asynchrony, but without a shift in the PSS, if exposed to desynchronized rather than synchronized audio-visual speech. Similar effects (a decrease in sensitivity after exposure to audio-visual asynchrony) were found when participants were exposed to audio-visual music stimuli. This led the authors to conclude that the window of temporal integration was widened because of asynchronous exposure (see also Navarra et al. 2007; and Winter et al. 2008 for effects on sensitivity after adaptation to asynchronous audio-tactile and motor-tactile stimuli, respectively). The authors argued that this widening may reflect an initial stage of recalibration in which a more lenient criterion is adopted for simultaneity. With prolonged exposure, participants may then ultimately shift the PSS. Alternatively, though, it might also be that participants became confused by the non-matching exposure stimuli in the background, and as a result made more errors, thus affecting the JND and not the PSS. Note that in the present study, the reduction in sensitivity after exposure to asynchronous videos was less likely caused by ‘confusion’ as such, because the exposure and test phase were clearly separated in time rather than that they were overlapping. However, given that the effect on the JND has most frequently been demonstrated in studies using naturalistic stimuli (i.e. hand motion, speech, music), it remains for future studies to examine whether the nature of the adapting stimuli (naturalistic vs. artificial) is critical for a widening of the temporal window.

An additional process that might be involved in the present study relates to the concept of *intentional binding* (Engbert et al. 2008; Haggard et al. 2002a, b; Moore et al. 2009; Blakemore et al. 2000; Engbert and Wohlschlager 2007; Cravo et al. 2009; Buehner and Humphreys 2009). It is commonly thought that intentional actions and their resulting effects are perceived as temporally attracted towards each other. In our study, it is therefore conceivable that the visual feedback was perceived as a consequence of a participant’s voluntary actions, and intentional binding may have contributed to a reduction of the perceived temporal delay. As a consequence, intentional binding may have led to an overall large proportion of ‘simultaneous’ responses, and sensitivity may for that reason be low. It should be noted, though, that from this perspective it is not clear how exposure to increasing delays—that presumably lead to *less* binding—could *worsen* sensitivity on test trials, because *less* binding should *improve*, not hamper sensitivity. In a similar vein, we observed that when the seen and felt location of the hands were aligned rather than misaligned—the latter evoking *less* binding—participants were in fact more (not less) sensitive to temporal asynchronies. Intentional binding thus cannot account for the differences in sensitivity when hands were aligned or when participants were exposed to large delays.

It is also of interest to note that equal shifts in the PSS were obtained when the virtual and real hand were aligned or misaligned. This fits an earlier study (Keetels and Vroomen 2007) in which participants were exposed to an asynchronous train of spatially matching or mismatching auditory-visual stimulus pairs. Following exposure to a fixed lag, participants were tested on an auditory-visual TOJ task. Temporal recalibration manifested itself as a shift of the PSS in the direction of the adapted auditory-visual lag. As observed here, this shift was equally big for spatially matching and mismatching sound/light pairs. Apparently, a spatial mismatch between otherwise corresponding inputs does not disrupt temporal recalibration. This is not to say, though, that spatial information is completely irrelevant for setting up the initial correspondence between inputs. As an example, in a study by Yarrow et al. (2011b), spatial correspondence resolved temporal ambiguity. Their participants were exposed to a train of sounds and lights whose locations alternated between left and right (e.g. left-sound, left-light, right-sound, right -light). The time between each stimulus was equal (200 ms), and in the absence of spatial information, it would be unclear whether sounds were actually leading or lagging the lights. The results though showed that temporal recalibration was obtained as implied by spatial grouping (in the previous example, sound-leading). This finding demonstrates that—in the absence of other cues—spatial information can help in the segmentation of the audio-visual scene.

A natural action like a finger tap is a complex stimulus, and this raises the question what the actual cues are in the signal that observers use as a timing marker for the action and for the visual feedback of that action. A finger tap might be decomposed into an intention to make a movement, followed by the actual motor command and an efferent copy of that command. While the finger is moving, the perceiver also receives proprioceptive feedback about the finger movement and the position of the joints, and tactile feedback at the moment that the finger touches an object. Conceivably, the visual feedback of the finger tap also contains several markers like the onset of the motion, the motion itself, and the offset of the visual motion when the finger touches the object. In the current situation, it is difficult to pinpoint which of these cues observers actually used to estimate the timing of the action and the feedback thereof. This requires further studies in which one could, for example, examine temporal recalibration with passive motion (to remove the intentional component), finger tapping without the touch on a surface (to remove the tactile feedback), or a restriction of the visual feedback to the onset or the offset of the finger tap.

Another relevant aspect of our study is that we asked participants to tap in synchrony with an auditory pacer (a click) presented at a constant rate (ISI = 750 ms). These isochronous clicks at a relatively slow rate ensured that consecutive finger taps were temporally distinct so that the timing relation with the video remained unambiguous (at faster rates, finger taps might overlap with the delayed video of the previous taps in which case the temporal relation would be lost). A typical outcome of auditory synchronization task is that finger taps precede the sound by about 20–60 ms (a ‘negative asynchrony’), most likely because the central representation of the tactile feedback of the finger is synchronized with the auditory code that represents the click. Because processing times are different for these two modalities, the tap has to lead the sound (for reviews, see Repp 2005; Aschersleben 2002). It remains for future studies to examine to what extent the presence of the auditory pacer was of help (e.g. serving as a timing anchor) for establishing the timing relation between the finger tap and its visual feedback.

Another question is to know what the maximum delay is to which the brain can still adapt. Of relevance is a study by Heron et al. (2009) that tested a substantially wider distribution of delays than used here (i.e. 50, 100, 200, 400, and 800 ms). In this study, participants pressed a mouse button five times. During the first four taps, an auditory, tactile, or visual stimulus was presented with a fixed delay (‘exposure phase’), and with the fifth mouse click, a sensory stimulus was presented with a variable delay (test stimulus). Participants judged whether the final stimulus appeared before or after the button click. Their results showed that the PSS was shifted as a function of exposure delays up to 200 ms, but then the effect declined for the 400 and 800 ms delays. The authors suggested that at the two largest delays, there was no ‘feeling of agency’, which then reduced recalibration. In the light of these findings, it is reasonable to assume that the 120-ms temporal delays used in the present study did not exceed the boundary for motor-visual unity. Further research is required, though, to examine whether the same criterion of agency holds for natural feedback and for other modalities like audition, as it may well be that for natural feedback (as in audio-visual speech) the criterion may wider.

Another relevant feature of our study is that we only used a relatively short-exposure phase of ten finger taps to induce temporal recalibration, while others have commonly used a single and longer exposure period of, for example, 3 min followed by short ‘top-up’ exposures (see Di Luca et al. 2009; Hanson et al. 2008; Vroomen et al. 2004; Keetels and Vroomen 2007, 2008; Fujisaki et al. 2004). In a recent study, Wozny and Shams (2010) also reported a fast recalibration process in the field of *spatial* recalibration (see also Frissen et al. 2012). In this study, it was shown that recalibration of sound location by a displaced visual stimulus did occur after just a single-exposure trial. This finding suggests that the modification of a sensory map may not necessarily require the accumulation of a substantial amount of evidence, but that it operates in a fast and continuous fashion. This fits with the observation that we obtained temporal recalibration after only a limited amount of exposure, though further testing is needed to examine the exact time course and whether there is accumulation across trials.

A related question is the extent to which temporal recalibration dissipates. Machulla et al. (2010) explored this by testing whether the strength of temporal recalibration decays over time or whether it declines due the presentation of new stimuli in the test phase. Their data showed that recalibration did not dissipate over time provided that no new sensory information was presented. Only when information was presented that differed from the stimuli used during adaptation, temporal recalibration diminished. Although the set-up of our study is not suitable for making strong claims about the time course of temporal recalibration, it seems safe to conclude that for the duration that the test phase lasted (i.e. five taps in total), and it did not completely undo the recalibration that was built-up during the relatively short-exposure phase (i.e. 10 taps).

To conclude, then, our results demonstrate that participants can adapt to a delay in the naturalistic visual feedback of a self-initiated motor action. This is remarkable, because the timing of visual feedback is in a natural situation usually without delay. While being exposed to delayed visual feedback, participants most likely adjust the point of subjective simultaneity (and sensitivity) of motor-visual synchrony, though other mechanisms may also be at play. Further research is required to examine the time course and the extent to which other modalities than vision can adjust the timing of naturalistic feedback.

## Footnotes

1 In many temporal perception studies, temporal order judgment (TOJ) tasks are used instead of simultaneity judgment (SJ) tasks (see Keetels and Vroomen 2012, p 149 for a discussion on both tasks). If a TOJ task was used in the present study, a participant would have to indicate whether the seen hand movement is earlier or later than the actual hand movement. Because participants know that it is practically impossible to see their hand moving before the movement has actually started, the use of a TOJ task would probably have led to a large bias towards ‘seen’ movement after ‘actual’ hand movement. No such bias plays a role in an SJ task, in which participants indicate whether the video was synchronous with the hand movement or not (both options are practically possible).

2 The intrinsic delay of the hardware was ~3 ms without noticeable variation. This value was measured via an oscilloscope with one sensor placed on the tapping device, and the other in the upper left corner of the screen. The build-up of the screen also took 20 ms (50 Hz). With the finger positioned at approximately half of the screen, it took ~13 ms (3 ms + 20/2 ms) to display the finger on the monitor. This 13 ms delay was constant for each condition and should be added to all delays mentioned here.
